# Research on the Relationship Between College Students’ Participation in Sports Activities and Self-Harmony Assessment Based on the Moderating and Mediating Effects of Mental Toughness

**DOI:** 10.3389/fpsyg.2022.919247

**Published:** 2022-06-06

**Authors:** Haixiao Xu

**Affiliations:** Physical Training Department, Hangzhou Medical College, Hangzhou, China

**Keywords:** college students, sports activities, self-harmony assessment, mental toughness, moderating and mediating effects

## Abstract

College students are the future of the motherland, the hope of the nation, and the reserve force to realize the great rejuvenation of the Chinese nation. The period of college students is an important period for the formation of ideals, beliefs, and world views. However, due to the contradictions between physiological maturity and psychological maturity and the contradictions between independent consciousness and cognitive ability in the growth process of college students, it is easy to cause internal psychological problems of inconsistency and disharmony. Correct sports lifestyle is conducive to promoting individual self-harmony. In the daily life and learning process of college students, the role of self-harmony is often ignored and not paid enough attention to. This indicates that it is necessary to further study the effect of physical activity on adolescents’ self-harmony. Mental toughness is one of the important factors affecting self-harmony. Therefore, the variable of mental toughness is introduced to analyze the correlation between physical activity and self-harmony. This study investigate the relationship between physical activity, mental toughness, and self-harmony in college students. Also this study can promote the re-recognition of college students of sports activities, explore the relationship between physical activity and self-harmony. Its purpose is to directly or indirectly promotes the college students’ mental health, and further enriches health psychology, exercise psychology, and development psychology-related theory and method. By analyzing the correlation among sports activities, mental toughness, and self-harmony, this study provides a theoretical reference for improving and enhancing the self-harmony degree of college students and providing beneficial suggestions for relevant management departments when making plans.

## Introduction

Mental toughness is embodied in different aspects, such as social adaptability, academic development, emotional adaptation, and so on. In addition, the effect of mental toughness in different fields is not the same. For example, college students show higher mental toughness in academic studies but lower mental toughness in social adaptability. A correct understanding of the connotation of college students’ mental toughness is conducive to a comprehensive understanding of the impact of college students’ mental toughness. The correlation between sports activities and mental health has been confirmed by more and more researchers. In the analysis of sports activities and mental health, many scholars say that sports activities can alleviate individual negative emotions, and sports activities can enhance individual self-confidence, optimism, tenacity, and other positive psychological qualities (Estrella et al., 2013; Stamp et al., 2017). Sports activities also have a promotion effect on the physiological level of the individual, specifically, they can improve the individual’s physical health and physical state and promote dopamine hormone secretion and other positive changes to promote the overall mental health level of the individual (Madrigal et al., 2013). Previous studies have also confirmed that sports activities have a positive role in promoting individual mental health, but relevant departments, schools, and society have not received corresponding attention, resulting in the neglect of physical education and individual mental health education in the process of physical education (Safian, 2020). Therefore, the interaction between sports activities and mental health should be emphasized in future educational practice (Gucciardi and Jones, 2012). At present, the psychological problems of college students are considered by researchers in various fields. Many college students commit suicide, feel weary, lack a yearning for life, and face other psychological problems frequently (Driska et al., 2012). This study mainly discusses the relationship among college students’ sports activities, mental toughness, and self-harmony. The purpose of studying the relationship among the three is to attract the attention of schools and parents and encourage college students to establish correct sports activities and establish correct lifelong sports awareness, which plays an irreplaceable role in improving college students’ mental health.

## Concept Introduction

### Sports Activity Participation

Research on sports activities has been interpreted differently by researchers in each field, and Michael (2012) defined that the concept of sports activities as the key to physical activity lies in physical means, improvement of physical and mental health, enrichment of material and cultural life, and other factors. Based on the combination of nature and hygiene, physical means are used to improve health and physique, develop physical quality, and regulate mental state. An activity aimed at enriching people’s cultural life and controlling their leisure time. Fen et al. (2015) believed that sports activities refer to planned, regular, and repeated sports activities under the interaction of their own conditions and external environment with the main purpose of enhancing physical fitness and improving physical and mental health. In this study, sports activities refer to all kinds of sports activities conducted by college students in accordance with their own needs and interests at school or outside school, as well as all sports activities arranged by the school curriculum, interclass exercise, and extracurricular activities. This study mainly investigates the sports activity items, sports activity time, sports activity frequency, and sports activity intensity of college students, so as to analyze the correlation among sports activity, mental toughness, and self-harmony.

### Self-Harmony

Bodroža (2011) argued that true self-harmony means that the self can freely follow the heart and choose what it wants to do, and the realization of true self-harmony must harmonize the real, realistic, and rational self in order to bring them into harmony and unity. According to Park et al. (2018), self-harmony refers to the ability of an individual to reconcile various contradictions and conflicts. If the ideal self or social self is far from the real self, it will lead to self-disharmony, inner conflict and tension, and psychological disorders. Fan et al. (2011) suggested that self-harmony refers to the consistency or closeness between self-knowledge and actual performance. In addition, good individual mentality, positive cognition, healthy psychology, optimistic spirit, and self-harmony promote each other. Guo (2013) believed that self-harmony means that the basic needs of self and external behavior can promote the personalized and social development of people and keep pace with the times to promote the construction of a harmonious society. In addition, the realization of self-harmony is of great significance to humans, nature, and society and is an important basis for promoting interpersonal, natural, and social harmony. This study adopts the definition of self-harmony given by Xiao (2013) and holds that the essence of self-harmony lies in self-satisfaction with the current life state or external environment, and being able to accept the cognition of internal and external environments of the body. Even if the individual’s own internal needs, self-set goals, and ideal results are not realized, or even the self-performance in all aspects is poor and difficult to surpass others, as long as the individual can accept the true self, without psychological obstacles and troubles, then he/she has achieved self-harmony.

### Mental Toughness

Mental toughness, also known as resilience, refers to an individual’s effective response and good adaptation to life pressure and setbacks (Mengmen and Suo, 2019). It belongs to the category of positive psychology, is a kind of positive psychological quality, and is a kind of ability that people can actively deal with difficulties and setbacks. Mental toughness is a bad resource, which can resist individual psychological disorders and behavioral problems. And the mental toughness has a positive impact on individual happiness. The improvement of college students’ mental toughness can not only help individuals improve their ability to overcome difficulties but also improve their life satisfaction and happiness levels and promote their mental health (Yasar and Turut, 2020). With the diversified development of society, people have higher and higher expectations and requirements of contemporary college students, which makes them bear great pressure in study and life, resulting in a series of psychological problems such as anxiety and depression. Life satisfaction is one of the important indicators to measure the mental health level of college students, and the improvement of life satisfaction is conducive to improving the mental health level. The increase of psychological resilience can effectively predict the increase of positive emotions and overall wellbeing (Gucciardi, 2011), and life satisfaction is one of the important dimensions of overall happiness, so psychological resilience may play an important role in the relationship between nostalgia and life satisfaction.

## Research Object and Methods

### Research Objects

The survey began in March 2021, and the research subjects were college students. Questionnaires were distributed to college students in the form of online electronic questionnaires, and 124 valid questionnaires were collected. Among them, there are 58 male students, accounting for 46.77%, and 66 female students, accounting for 53.23%. The basic information of the research object is shown in Table 1.

**TABLE 1:** Basic information of the research subject.

Statistical variables of the sample		Number of the sample	Proportion (%)
Grade	Freshman	39	31.45
	Sophomore	41	33.06
	Junior year	21	16.94
	Senior year	23	18.55
Gender	Male	58	46.77
	Female	66	53.23
Origin of student	City	36	29.03
	Villages and towns	56	45.16
	Rural	32	25.81

### Research Methods

#### Sports Activity Participation Measurements

According to Liang (1994), the Physical Activity Compile Physical Activity Rating Scale (PARS-3) measures the amount of exercise from three aspects, which are sports activity time, sports activity frequency, and sports activity intensity. Each item has five different levels of options from low to high. The specific calculation formula is as follows:

Exercise Score = Intensity × (time−1) × Frequency

#### Self-Harmony Measurement

According to the Self-harmony Rating Scale compiled by Wang and Cui (2007), the scale includes three subscales: “the disharmony between self and experience,” “the self-flexibility,” and “the self-rigidity.” There are a total of 35 items on the scale, and each item is divided into five grades and recorded as 1–5 points, respectively. The total score of self-harmony can be calculated. Specifically, “the self-flexibility” is scored in reverse and then added to the scores of the other two subscales.

#### Mental Toughness Measurement

Mental toughness is compiled by Dagnall (mental toughness questionnaire-10, MTQ-10). The scale has a total of 10 items, which are optimized by the author and selected as follows: objective focus, emotion control, positive cognition, family support, and interpersonal assistance were measured from 1 (strongly disagree) to 5 (strongly agree). The higher the score, the better the individual’s psychological resilience (Driska et al., 2012). MTQ-10 was used in this study, and Cronbach’s α coefficient of consistency reliability of the scale was 0.72, greater than 0.7, indicating good reliability and reliable measurement results.

### Research Hypothesis

Jackman et al. (2017) pointed out that for college students to have a better understanding of themselves, they must face some setbacks in the process of engaging in sports activities, find themselves from setbacks, better play their potential, and better adapt to setbacks, pressure, and negative emotions in a better and faster manner. This study preliminarily analyzes the research results on the relationship among sports activities, mental toughness, and self-harmony of college students and proposed the following five research hypotheses:

Hypothesis 1: There are significant differences in demographic variables among sports activities, mental toughness, and self-harmony among college students.
Hypothesis 2: There is a significant positive correlation between sports activities and mental toughness of college students.
Hypothesis 3: There is a significant negative correlation between sports activities and self-harmony of college students.
Hypothesis 4: There is a significant negative correlation between college students’ mental toughness and their self-harmony.
Hypothesis 5: Mental toughness plays a mediating role in the correlation between sports activities and self-harmony.

### Statistical Method

SPSS 22.0 software was used to analyze the data, including descriptive statistics, one-way variance, independent sample *t*-test, internal consistency test, multiple linear regression analysis, and path analysis.

## Results

### Demographic Results

To better understand the relationship among college students’ sports activities, mental toughness, and self-harmony. Descriptive statistics were made on the relationship among sports activities, mental toughness, and self-harmony among college students. Demographic results are shown in Table 2.

**TABLE 2:** Demographic results.

First-level indicator	Second-level indicator	Average	Standard deviation
Sports activities participation	Sports activity intensity	12.32	5.45
	Sports activity time	23.15	1.76
	Sports activity frequency	23.29	1.85
Self-harmony	Disharmony between self and experience	23.75	1.59
	Self-flexibility	22.82	13.26
	Self-rigidity	53.21	8.05
Mental toughness	Objective focus	95.15	9.68
	Emotion control	46.25	13.26
	Positive cognition	28.18	9.66
	Family support	19.73	7.28
	Interpersonal assistance	52.01	13.18

### Difference Between Sports Activity Participation and Mental Toughness and Self-Harmony

One-way ANOVA was conducted for the mental toughness, factors, and self-harmony of college students with different sports activities. The results show that there are significant differences between college students and college students’ mental toughness. There is a significant correlation between sports activity time and objective focus, emotion control, positive cognition, and interpersonal assistance, with a correlation coefficient <0.05. There is no significant correlation between sports activity time and family support. Therefore, there is a significant positive correlation between sports activity time and objective focus, emotion control, positive cognition, and interpersonal assistance.

The sports activity frequency of college students is significantly correlated with objective focus, emotion control, positive cognition, and family support, with the correlation coefficient reaching <0.05. There is no significant correlation between sports activity frequency and interpersonal assistance (*P* > 0.05). Therefore, there is a significant positive correlation among sports activity frequency and objective focus, emotion control, positive cognition, and family support, exercise intensity, exercise time, and exercise frequency, and there are significant differences between college students and college students’ mental toughness. The difference between sports activities participation and mental toughness and self-harmony is shown in Table 3.

**TABLE 3:** Difference between sports activity participation and mental toughness and self-harmony.

Factor	Sports activity intensity	Sports activity time	Sports activity frequency	*F*
Objective focus	17.25 ± 3.51	18.81 ± 3.50	18.77 ± 3.52	2.180
Emotion control	18.55 ± 4.50	20.24 ± 4.55	20.19 ± 5.04	2.446
Positive cognition	14.79 ± 2.72	16.08 ± 2.68	16.28 ± 3.01	1.135
Family support	20.70 ± 4.96	21.33 ± 2.96	22.17 ± 4.46	1.128
Interpersonal assistance	19.24 ± 5.18	20.20 ± 5.49	20.24 ± 5.28	0.769
Disharmony between self and experience	45.65 ± 8.36	44.53 ± 9.35	44.58 ± 9.70	1.308
Self-flexibility	44.28 ± 5.48	45.73 ± 5.64	45.52 ± 6.23	1.147
Self-rigidity	18.59 ± 4.17	18.09 ± 3.89	18.27 ± 4.39	0.947

### Correlation Matrix Between Sports Activity Participation and Mental Toughness and Self-Harmony

There is a significant correlation between college students’ sports activity intensity and objective focus, emotion control, and positive cognition, with a correlation coefficient of <0.05. There is no significant correlation between sports activity intensity and family support and interpersonal assistance (*P* > 0.05). Therefore, there is a significant positive correlation between sports activity intensity and objective focus, emotion control, and positive cognition.

There is a significant correlation between sports activity time and objective focus, emotion control, positive cognition, and interpersonal assistance, with a correlation coefficient of <0.05. There is no significant correlation between sports activity time and family support. Therefore, there is a significant positive correlation between sports activity time and objective focus, emotion control, positive cognition, and interpersonal assistance.

The sports activity frequency of college students is significantly correlated with objective focus, emotion control, positive cognition, and family support, with the correlation coefficient reaching <0.05. There is no significant correlation between sports activity frequency and interpersonal assistance (*P* > 0.05). Therefore, there is a significant positive correlation between sports activity frequency and objective focus, emotion control, positive cognition, and family support. The correlation matrix between sports activity participation and mental toughness and self-harmony is shown in Table 4.

**TABLE 4:** Correlation matrix between sports activity participation and mental toughness and self-harmony.

Factor	Objective focus	Emotion control	Positive cognition	Family support	Interpersonal assistance
Sports activity intensity	0.136	0.128	0.068	0.028	0.026
Sports activity time	0.223	0.165	0.071	0.036	0.082
Sports activity frequency	0.169	0.132	0.114	0.068	0.046

There is no significant correlation between college students’ sports activity intensity and disharmony between self and experience and self-rigidity (*P* > 0.05). There is a significant correlation between sports activity intensity and self-flexibility of college students, and the correlation coefficient is <0.05. There is a significant correlation between sports activity time and disharmony between self and experience and self-flexibility, and the correlation coefficient is <0.05. There is no significant correlation between sports activity time and self-rigidity (*P* > 0.05). There is a significant correlation between the sports activity frequency of college students and disharmony between self and experience and self-flexibility, and the correlation coefficient is <0.05. There is no significant correlation between sports activity frequency and self-rigidity (*P* > 0.05). Therefore, there is a significant negative correlation between the sports activity frequency and the disharmony between self and experience and a positive correlation between the sports activity frequency and the self-flexibility. The correlation matrix between college students’ sports activities participation and self-harmony is shown in Table 5.

**TABLE 5:** Correlation matrix between college students’ sports activity participation and self-harmony.

Factor	Disharmony between self and experience	Self-flexibility	Self-rigidity
Sports activity intensity	−0.028	0.065	−0.029
Sports activity time	−0.112	0.121	0.023
Sports activity frequency	−0.071	0.120	0.015

There is a significant correlation between college students’ mental toughness and disharmony between self and experience, self-flexibility, and self-rigidity, with the correlation coefficient <0.05. Moreover, there is a significant negative correlation between mental toughness and disharmony and between self and experience, self-rigidity, and positive correlation with self-flexibility, that is, the higher the score of mental toughness, the lower the score of disharmony between self and experience and self-rigidity, and the higher the score of self-flexibility. In other words, college students with a high level of mental toughness have a lower degree of disharmony between self and experience, a lower degree of self-rigidity, a higher degree of self-flexibility, and a higher degree of self-harmony. The correlation matrix between college students’ mental toughness and self-harmony is shown in Table 6.

**TABLE 6:** Correlation matrix between college students’ mental toughness and self-harmony.

Factor	Objective focus	Emotion control	Positive cognition	Family support	Interpersonal assistance
Disharmony between self and experience	−0.234	−0.536	−0.078	−0.271	−0.465
Self-flexibility	0.432	0.208	0.369	0.215	0.246
Self-rigidity	−0.118	−0.204	−0.123	−0.175	−0.317

### Regression Analysis of Sports Activity Participation and Mental Toughness and Self-Harmony

#### Regression Analysis of Sports Activity Participation on Mental Toughness

Sports activity intensity, time, and frequency are the independent variables, mental toughness total score is the dependent variable, regression equation model is established, the selection of mental toughness of most prediction model is analyzed, stepwise regression method is used for analysis, and *F*-test is used to screen whether variables are included in the regression equation. *P* < 0.05 is included in the regression equation, and *P* > 0.1 is excluded from the regression equation. As shown in Table 7, only the time and frequency of sports activity enter the regression equation, and a model is established with a coefficient of determination of 0.206. The explanation of physical activity for mental toughness is 4.3%, and the variance test also reaches the significance level. The regression analysis of sports activity participation on mental toughness is shown in Table 7.

**TABLE 7:** Regression analysis of sports activity participation on mental toughness.

Factor	Standard regression coefficient	*t*	*R*	*R* ^2^	Adjusted *R*^2^	*F*
Sports activity time	0.135	3.854				
Sports activity frequency	0.104	2.713	0.206	0.043	0.047	22.563

#### Regression Analysis of Sports Activity Participation on Self-Harmony

With the total score of sports activities and each factor as an independent variable and self-harmony as a dependent variable, the step regression method is used for analysis, *F*-test is used as the criterion to screen whether variables are included in the regression equation, *P* < 0.05 is included in the regression equation, and *P* > 0.1 is removed from the regression equation. The results are shown in Table 8. Physical activity intensity is entered into the regression equation, and a model is established. The determination coefficient of the model is 0.113, the amount of physical activity explaining self-harmony is 1.7%, and the variance test reaches the significance level. The regression analysis of sports activity participation on self-harmony is shown in Table 8.

**TABLE 8:** Regression analysis of sports activity participation on self-harmony.

Factor	Standard regression coefficient	*t*	*R*	*R* ^2^	Adjusted *R*^2^	*F*
Sports activity intensity	−0.105	−3.414	0.113	0.017	0.013	12.063

#### Regression Analysis of Mental Toughness to Self-Harmony

Mental toughness total score and each factor score are independent variables, the self-harmony total score is the dependent variable, which is analyzed with the method of stepwise regression analysis, and the *F*-test is used as a standard of whether screening of variables into the regression equation, *P* < 0.05 is into the regression equation, *P* > 0.1 is in eliminating regression equation, and the selection of self-harmony of the most prediction model are studied. The results are shown in Table 9. Objective focus, positive cognition, and family support enter the regression equation, and a model is established. The coefficient of determination of the model is 0.563, and the amount of explanation of resilience to self-harmony is 45.6%. The regression analysis of mental toughness to self-harmony is shown in Table 9.

**TABLE 9:** Regression analysis of mental toughness to self-harmony.

Factor	Standard regression coefficient	*t*	*R*	*R* ^2^	Adjusted *R*^2^	*F*
Objective focus	0.062	2.312				
Positive cognition	0.069	2.563				
Family support	−0.812	−18.926	0.563	0.456	0.423	203.328

### Mediating Effect Test

#### Analysis of Various Model Indicators

The indicators all meet the critical value, indicating that the research model has a good degree of fit. From the model analysis, the CMIN is equal to 63.158, and the *P*-value is equal to 0.000 (*P* < 0.005), indicating that there is a significant difference between the variance matrix derived from path analysis and the variance matrix derived from the hypothetical model; and the overall fitting index of the model meets the basic requirements, so the model established in this study conforms to the path analysis, and the model is accepted. The fitting index of each model index is shown in Table 10.

**TABLE 10:** Fitting index of each model index.

Model indexes	Model fitting index before modification	Model fitting index after modification	Critical value
CMIN	341.208	63.158	
CMIN/DF	8.506	2.084	
IFI	0.845	0.936	>0.90
CFI	0.847	0.925	>0.90
NFI	0.836	0.928	>0.90
GFI	0.925	0.974	>0.90
RFI	0.752	0.936	>0.90
AGFI	0.896	0.964	>0.90
PNFI	0.602	0.534	>0.50
PGFI	0.557	0.436	>0.50
RMSEA	0.086	0.037	<0.05

#### Path Factor Analysis

**FIGURE 1:**
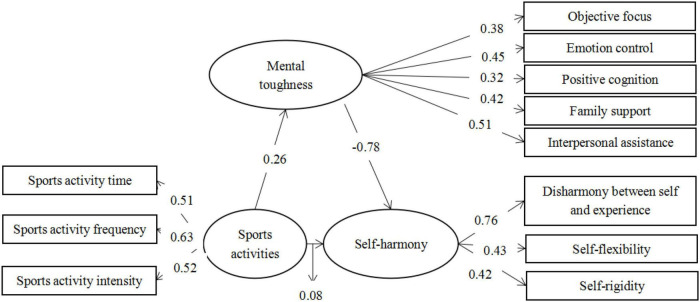
Path analysis of intermediate variables.

The fitting degree of each model index in this study is good, so the path coefficients among physical activity level, mental toughness, and self-harmony are all meaningful. Amos was used to calculate the standardized path coefficients between each path (Parkes and Mallett, 2011). Results from the path output showed that the factor load *P*-value is less than 0.001, except for the physical activity on the self-harmony between the path factor *P*-value is greater than 0.05. The standardized path coefficient of sports activities to mental toughness is 0.26, the standardized path coefficient of sports activities to self-harmony is 0.08, and the standardized path coefficient of mental toughness to self-harmony is −0.78. Path analysis of intermediate variables is shown in Figure 1.

#### Mediating Effect Analysis

The Amos test equation model is used to output the results. To analyze whether mental toughness is a partial intermediary or a complete intermediary in the impact of sports activities on self-harmony in this study, Amos was used to conduct path analysis, Bootstrap is used to calculate standardized estimates and standard deviations, and the results are analyzed according to the output results of the path (Mahoney et al., 2014). Therefore, mental toughness plays a complete mediating role in the relationship between sports activities and self-harmony. Mediating effect analysis results are shown in Table 11.

**TABLE 11:** Mediating effect analysis results.

Coefficient name	Normalized path coefficients	Standard error	Significant or not
a	0.265	0.048	Yes
b	−0.816	0.049	Yes
C	−0.153	0.067	Yes
c’	0.054	0.039	No

**FIGURE 2:**
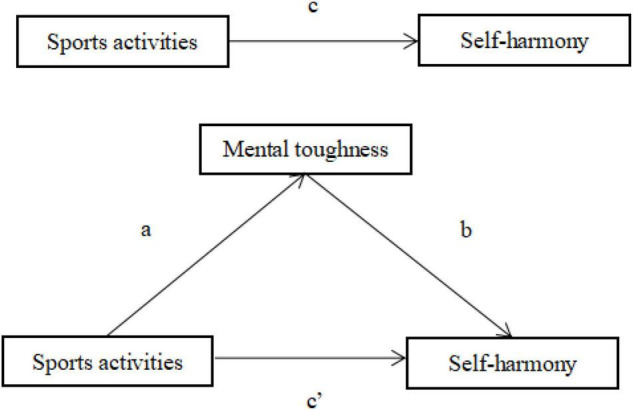
Mediation equation model.

The mediation equation model is shown in Figure 2.

The total effect value of physical activity on self-harmony is −0.152, which is also the sum of the direct effect value and the indirect effect value. The direct effect value is 0.049, and the indirect effect value is −0.209. The direct path of physical activity to self-harmony is not significant, while the indirect path of sports activities to self-harmony is significant through mental toughness. According to the bootstrap mediation test method, there are two confidence intervals in the path analysis result report, namely, the bias-corrected percentile method and the percentile method. The bias correction confidence interval is used to observe the influence of physical activity on self-harmony. In this study, the lower and upper limits of total effect and indirect effect do not contain 0, while the confidence interval of direct effect contains 0. The total effect and indirect effect of sports activities on self-harmony are significant, while the direct effect of sports activities on self-harmony is insignificant. It also confirms that mental toughness played a completely mediating role in the relationship between sports activities and self-harmony. The indirect effect accounted for 63.15% of the total effect. The path analysis results are shown in Table 12.

**TABLE 12:** Path analysis results.

Path name	Normalized path coefficient	Ratio of total effect (%)	Upper limit	Lower limit	Significant or not
Direct effect	0.049	36.52	0.129	−0.025	No
Indirect effect	−0.209	63.15	−0.130	−0.286	Yes
Total effect	−0.152	−	−0.059	−0.234	Yes

## Research Conclusion

University is a relatively complex small society, and the ability of college students to adapt to this small society depends on their mental toughness to a certain extent. Mental toughness is a buffer for college students to face setbacks and failures, and it is a kind of resilience ability in life pressure and frustration. College students with high mental toughness can better overcome adversity and setbacks and pursue positive self-realization. College students who regularly participate in sports activities have a higher level of mental toughness than those who do not participate in sports activities. Meanwhile, sports activities can directly affect the level of the mental toughness of college students, and sports activities can also indirectly affect the level of mental toughness by affecting other psychological variables. The conclusions of this study include the following:

(1) At present, most of the physical activities of college students belong to a small amount of exercise. The amount of physical activity of college students has a significant difference in gender, grade, and origin. In terms of scores, freshmen and sophomores are higher than juniors and seniors, and cities are higher than towns and villages.
(2) The degree of college students’ mental toughness is above average, and there is a significant difference in the origin of college students’ mental toughness. In terms of scores, cities are higher than towns and villages. The degree of self-harmony of college students is medium and high, and there are significant differences in the grade and origin of students. The degree of self-harmony of college students in cities is higher than that in towns and villages.
(3) Sports activities have a direct impact on college students’ mental toughness. There are significant differences in exercise intensity, exercise time, exercise frequency, and exercise quantity, and there is a significant positive correlation between sports activities and mental toughness.
(4) There is a positive influence between sports activities and college students’ self-harmony. There are significant differences in the time and frequency of exercise among college students, but no significant differences in the intensity and amount of exercise. There is a significant correlation between intensity, time, frequency, physical activity, and self-harmony.
(5) The direct path of sports activities on college students’ self-harmony is not significant, but the indirect path of sports activities on college students’ self-harmony is significant through enhancing the level of mental toughness. Therefore, mental toughness is a complete mediator between sports activities and self-harmony, and the indirect effect is 63.15% of the total effect.

The innovation of this study include: by investigating college students’ sports activities, the relationship between mental toughness and self-harmony promotes the re-recognition of college students of sports activities; this study further explores the relationship between physical activity and self-harmony, and finds that sports activities can directly or indirectly promotes the college students’ mental health. Furthermore, our study have found that sports activities can greatly enrich the relevant theories and methods of health psychology, sports psychology, and developmental psychology. However, due to the constraints of time and funds in the process of research and investigation, the number of questionnaires issued is relatively small. In effective questionnaires, the sample size is relatively small, and 124 valid questionnaires were collected. But there are certain limitations in the area of investigation, which may not represent the overall situation of college students in China. In related research in the future, it is better to conduct a survey on the sports activities of national college students, so that the results of this study can be more representative. Further attention of relevant departments must be aroused to better strengthen the mental health education of college students.

## Data Availability Statement

The original contributions presented in the study are included in the article/supplementary material, further inquiries can be directed to the corresponding author.

## Ethics Statement

Ethical review and approval was not required for the study on human participants in accordance with the local legislation and institutional requirements. The patients/participants provided their written informed consent to participate in this study.

## Author Contributions

HX contributed to wiring, data collection, and methodology.

## Conflict of Interest

The author declares that the research was conducted in the absence of any commercial or financial relationships that could be construed as a potential conflict of interest.

## Publisher’s Note

All claims expressed in this article are solely those of the authors and do not necessarily represent those of their affiliated organizations, or those of the publisher, the editors and the reviewers. Any product that may be evaluated in this article, or claim that may be made by its manufacturer, is not guaranteed or endorsed by the publisher.
